# Utilization of ancient permafrost carbon in headwaters of Arctic fluvial networks

**DOI:** 10.1038/ncomms8856

**Published:** 2015-07-24

**Authors:** Paul J. Mann, Timothy I. Eglinton, Cameron P. McIntyre, Nikita Zimov, Anna Davydova, Jorien E. Vonk, Robert M. Holmes, Robert G. M. Spencer

**Affiliations:** 1 Department of Geography, Northumbria University, Newcastle Upon Tyne NE1 8ST, UK; 2 Department of Earth Sciences, Geological Institute, ETH Zürich, 8092 Zürich, Switzerland; 3 Department of Physics, Laboratory for Ion Beam Physics, ETH Zürich, 8093 Zürich, Switzerland; 4 North-East Science Station, Pacific Institute for Geography, Far-Eastern Branch of Russian Academy of Science, Cherskiy, Republic of Sakha (Yakutia), Russia; 5 Department of Earth Sciences, Utrecht University, Heidelberglaan 2, 3584 CS Utrecht, The Netherlands; 6 Arctic Centre, University of Groningen, PO Box 716, 9700 AS Groningen, The Netherlands; 7 Woods Hole Research Center, Falmouth, Massachusetts 02540, USA; 8 Department of Earth, Ocean and Atmospheric Science, Florida State University, Tallahassee, Florida 32306, USA

## Abstract

Northern high-latitude rivers are major conduits of carbon from land to coastal seas and the Arctic Ocean. Arctic warming is promoting terrestrial permafrost thaw and shifting hydrologic flowpaths, leading to fluvial mobilization of ancient carbon stores. Here we describe ^14^C and ^13^C characteristics of dissolved organic carbon from fluvial networks across the Kolyma River Basin (Siberia), and isotopic changes during bioincubation experiments. Microbial communities utilized ancient carbon (11,300 to >50,000 ^14^C years) in permafrost thaw waters and millennial-aged carbon (up to 10,000 ^14^C years) across headwater streams. Microbial demand was supported by progressively younger (^14^C-enriched) carbon downstream through the network, with predominantly modern carbon pools subsidizing microorganisms in large rivers and main-stem waters. Permafrost acts as a significant and preferentially degradable source of bioavailable carbon in Arctic freshwaters, which is likely to increase as permafrost thaw intensifies causing positive climate feedbacks in response to on-going climate change.

## Abstract (web summary)

The climatic impact of ancient carbon released during the thawing of Arctic permafrost depends on the degree to which it is degraded. Here, the authors show that permafrost-sourced carbon is preferentially metabolized by microbial communities during transit in high-latitude rivers.

## Graphical abstract

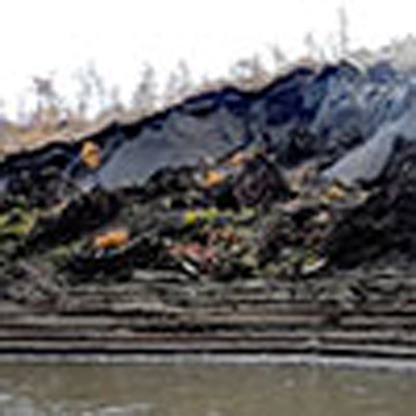

Climate-induced Arctic warming has led to increased soil temperatures, causing a succession of changes associated with permafrost degradation and widespread ground collapse or thermokarst, including deepening of the seasonally thawed surface active layer^1^,^2^ and alterations to watershed hydrology^3^,^4^. These changes threaten to destabilize ancient (Pleistocene-aged) northern circumpolar terrestrial permafrost, which contains vast quantities of organic carbon (OC) comprising as much as 50% of global below-ground soil carbon stocks^5^. Consequently, soil OC losses of up to 220 PgC by 2100 are predicted across Arctic regions^6^,^7^, leading to large-scale mobilization of permafrost-derived OC into Arctic inland and coastal waters^8^.

The great majority of OC (>80%)^9^ supplied to coastal seas from Arctic rivers is in the form of dissolved OC (DOC) and thus readily available to support microbial carbon demand during transit through fluvial networks^10^. Growing evidence exists from temperate and sub-tropical regions^11^,^12^,^13^ that older aged terrestrial carbon can be an important subsidy to microbial communities, yet the processing and fate of ancient Arctic permafrost OC throughout fluvial networks is currently unknown. Understanding the response of aquatic systems to future increases in permafrost-derived DOC is necessary for determining its effects upon global carbon cycling and predicting its export via high-latitude rivers.

Studies from major Arctic rivers show that higher-order fluvial systems contain predominantly young (^14^C-enriched) DOC^14^,^15^,^16^,^17^, suggesting that limited mobilization of permafrost has occurred to date or that it has been removed upstream. A number of studies focusing on lower-order and headwater streams provide evidence for ancient DOC mobilization^18^,^19^. Elucidating the fate of permafrost-derived DOC is crucial for establishing if on-going thaw is currently impacting Arctic fluvial networks and in determining whether its turnover may result in a positive carbon feedback to climate change^7^.

Here, we examine the quantity, age and source of the OC present, and constrain the nature of OC supporting microbial carbon demand with increasing water residence time through fluvial networks (higher order in the stream network). We base this work in the Kolyma River Basin, Siberia, which represents the largest global watershed (ca. 650,000 km^2^) completely underlain by continuous permafrost. The majority of this permafrost is comprised of frozen Yedoma, organic-rich (1–5% carbon by mass) Pleistocene-aged deposits that can contain 50–90% ice content^5^,^20^. Yedoma–ice complexes measured in the Kolyma Basin suggest that permafrost carbon by mass concentrations are on the order of 1.5±1.4%, with an average carbon inventory amounting to 14±8 kg m^−3^ (refs 21, 22, 23). Thawing Yedoma-derived OC is thought to be highly susceptible to biological degradation in fluvial networks^19^,^24^, and has been proposed as a key feedback upon global climate^20^.

We first assess the initial age and bioavailability of DOC in streams and rivers throughout the basin using measurements of biologically degradable DOC (using DOC loss) and ^14^C-DOC measurements. We then determine the source and age of DOC subsidizing microbial demand throughout the network using simultaneous measurements of stable and radiocarbon signatures of DOC (^13^C and ^14^C) during short-term (28 days) bioincubation experiments. We show that a preferential loss of permafrost-derived DOC occurs with increasing water residence and inland water transit time, despite similar initially modern DOC ages. Our results demonstrate that microbial communities selectively remove ancient OC during water transit throughout the fluvial network, with a potential to rapidly act as a positive feedback upon climate change with increasing permafrost thaw.

## Results

### Initial ^14^C-DOC age and proportion bioavailable DOC

**Figure 1:**
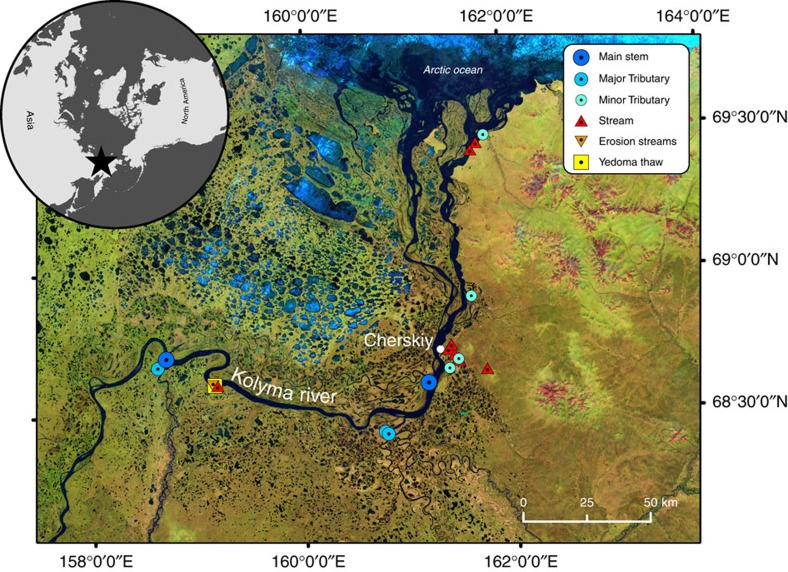
Study site and sample locations. Location and type of waters collected throughout the fluvial network. Cherskiy and site of the Northeast science station is marked in white. Individual site latitude and longitude information is provided in Supplementary Table 1.

**Figure 2:**
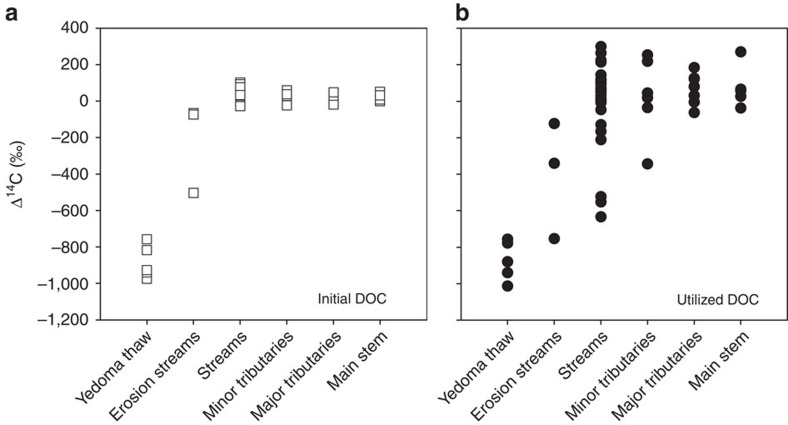
Downstream Δ^14^C DOC values. Δ^14^C values of (**a**) initial DOC, and (**b**) utilized DOC supporting microbial demand (DOC_loss_) during bioincubations. Δ^14^C values of utilized DOC were calculated as outlined in equation (1) (see Methods). Table 1 displays mean values for initial and utilized DOC across sites and sample numbers for each site type.

We conducted 81 individual incubation experiments on samples from 19 streams and rivers throughout the Kolyma fluvial network, grouping by stream size and type (Fig. 1; Supplementary Table 1). Thaw streams, directly draining Yedoma outcrops, contained extremely high concentrations of DOC (10,939±1,278 μM) that are highly depleted in ^14^C (Δ^14^C−883±41‰; Fig. 2a). A large proportion of thaw stream DOC was utilized by microorganisms (DOC_loss_) during short-term (28 days, 20 ^°^C) incubations (47±8%), confirming the high bioavailability of permafrost-derived carbon in fluvial systems^19^,^24^ (Table 1; Supplementary Table 2). Mean DOC concentrations declined moving downstream through the fluvial network, yet proportions of DOC_loss_ were similar (15–21%) among all other stream and river waters (analysis of variance *P*>0.05, Table 1; Supplementary Table 2). Erosion-impacted streams that receive greater amounts of permafrost soil inputs via mechanical erosion of the stream banks contain ^14^C-depleted DOC (−214±145‰; Fig. 2a), highlighting the strong potential for thermokarst processes to sporadically deliver aged OC to fluvial networks. By contrast, all other stream and river waters contained modern initial bulk DOC pools (Δ^14^C>−50‰; Fig. 2a), consistent with the majority of available radiocarbon data for DOC from major Arctic Rivers^14^,^15^,^18^.

### Radiocarbon age of DOC supporting microbial demand

Microbial communities in Yedoma thaw stream waters consistently utilized highly ^14^C-depleted DOC (−750 to −1,000‰), indicating that Pleistocene-age OC (11,300 to >50,000 ^14^C years) is intrinsically biologically labile and immediately available for microbial metabolism after mobilization to freshwaters (Fig. 2b). Microbial demand in erosion-impacted streams was also supported by highly aged DOC (Δ^14^C, −405±185‰, Table 1), demonstrating that microbial communities in waters containing mixed modern and ancient OC pools can preferentially utilize an older OC fraction (up to 10,000 ^14^C years). Furthermore, despite similar modern initial bulk DOC ages among other stream and river waters, Δ^14^C values of the DOC utilized by microorganisms across these sites (that is, the fraction consumed during bioincubations) varied considerably (Δ^14^C+300 to −630‰; Fig. 2b), indicating a disconnect between bulk DOC age and the age of DOC fuelling microbial demand^11^,^13^. Modern (^14^C-enriched) DOC periodically utilized by microorganisms through the river network may have been delivered from soil pore waters that can contain high values of Δ^14^C-DOC^25^ or may be caused by the upstream preferential loss of aged OC.

### Source and radiocarbon age of DOC supporting microorganisms

**Figure 3:**
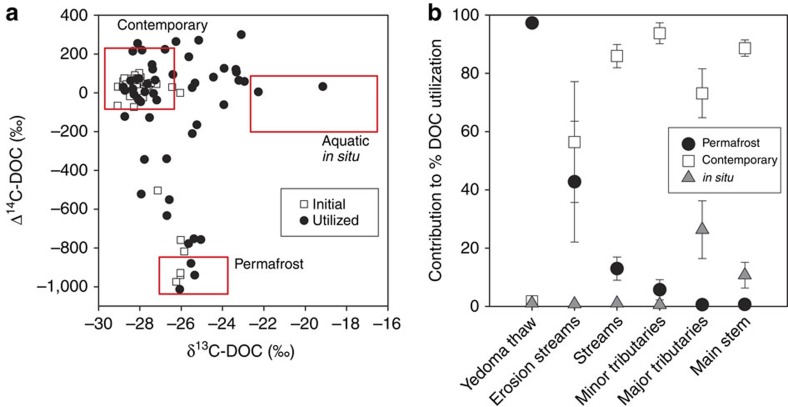
Distribution of ^14^C and ^13^C isotopes and end-member contributions across the fluvial network. (**a**) Initial (open squares) and utilized (black circles) Δ^14^C and δ^13^C values of DOC. Isotopic ranges of three contributing carbon sources are shown in red boxes (see Methods). (**b**) Mean percent (±s.e.m.) contribution of each carbon source to the DOC utilized over bioincubations (DOC_loss_) in waters from differing site types.

We used a dual-isotope approach^26^ to examine the source and age of OC subsidizing DOC_loss_ throughout the stream network. We partitioned source contributions that most likely explained utilized Δ^14^C and δ^13^C values of DOC_loss_, assuming three dominant sources of DOC (contemporary terrestrial OC, permafrost-derived terrestrial OC and internally produced *in situ* OC) (Fig. 3a).

Microbial demand in Yedoma thaw waters was almost entirely (97.2±0.8%) supported by permafrost-derived OC, demonstrating that ancient DOC was highly bioavailable when present. Downstream, permafrost subsidized greater proportions of DOC_loss_ in higher-order erosion-impacted (42.8±20.7%) and other small streams (13.0±4.0%) as compared with minor tributary (5.7±3.5%), major tributary (0.6±0.1%) and main-stem waters (0.7±0.1%) (Table 1). This landscape pattern of declining permafrost contribution to DOC_loss_ through the fluvial network proved consistent across both years of investigation (2012 and 2013), showing that microbial demand in thaw streams and headwaters is consistently subsidized by significant proportions of permafrost OC. The fact that DOC character and age altered with increasing water residence time does not simply reflect catchment variability, as evidenced by identical patterns of DOC modernization in sub-basins within the larger Kolyma watershed. Furthermore, most sites drained similar soil and vegetation types, and spanned little elevation change within the Kolyma lowlands. Utilized Δ^14^C of DOC_loss_ increased exponentially (*r*^2^=0.72, *P*<0.001, Supplementary Fig. 1) relative to initial Δ^14^C values, suggesting that older OC fractions were rapidly and preferentially mineralized when present in bulk DOC (initial Δ^14^C<−20‰). Terrestrially derived contemporary OC contributed towards a minor proportion of DOC_loss_ in thaw waters (1.7±0.5%), but comprised the main OC source (>55%; Table 1) across stream and river waters, emphasizing the dominance of surface soil–water dynamics in determining bulk DOC character and thus biogeochemical processing and fate (Fig. 3b).

### Environmental and compositional effects on ^14^C utilization

**Figure 4:**
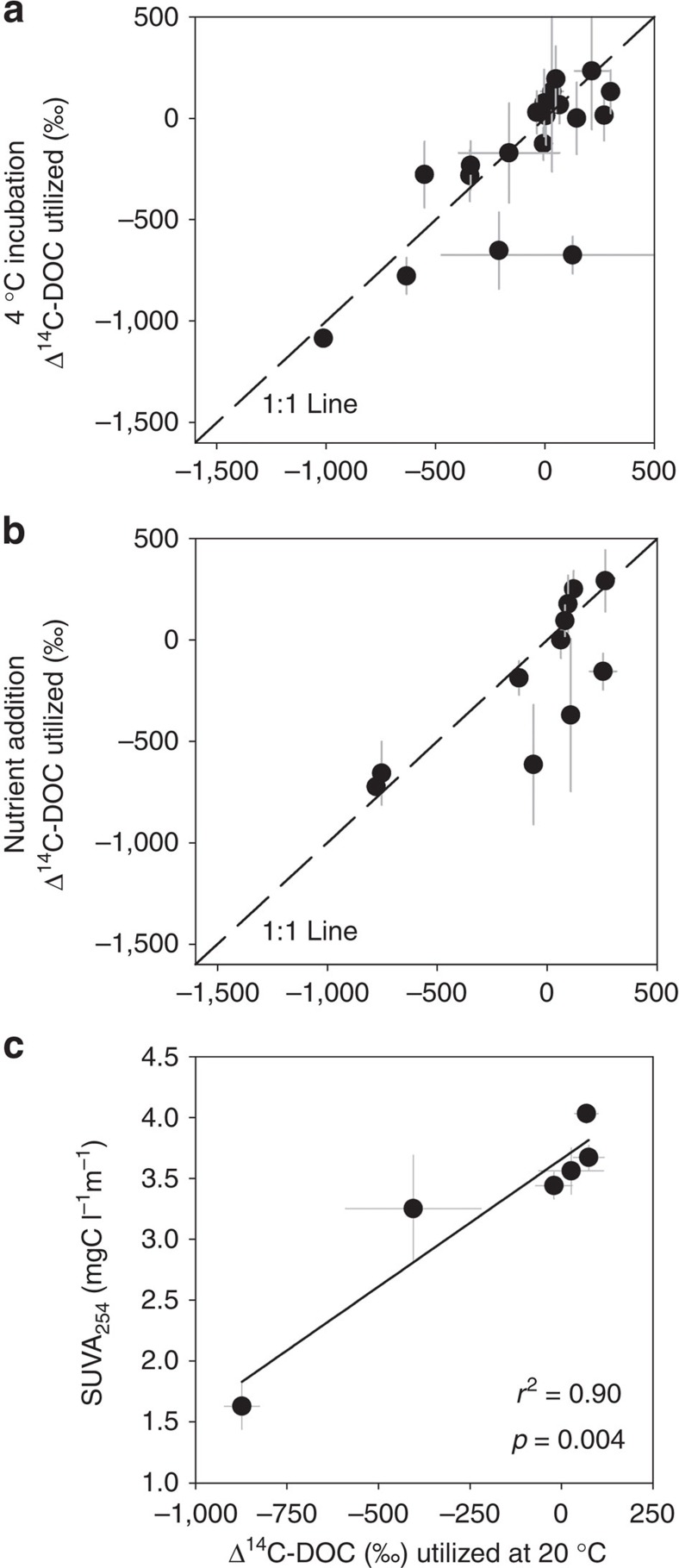
Environmental and OC compositional controls on the age of carbon supporting freshwater microbial demand. Δ^14^C values of DOC utilized (±propagated s.e.m.) in parallel incubations conducted (**a**) at 20 and 4 ^°^C (*n*=19) and (**b**) under ambient and nutrient-enriched conditions (*n*=11). Dashed line represents 1:1 line. (**c**) Δ^14^C of mean DOC utilized across sites in relation to initial DOC aromaticity (inferred from SUVA_254_, see text for details). Decreasing SUVA_254_ indicates declining DOC aromaticity^34^.

Seasonal and spatial trends in OC bioavailability across fluvial networks can be controlled by variations in water temperature, inorganic nutrient availability and OC character^24^,^27^,^28^,^29^,^30^. To examine whether downstream variations in water temperature and inorganic nutrient concentrations influenced the fraction and age of DOC utilized, we conducted parallel experiments incubating waters at 4 ^°^C and under nutrient-replete conditions. Total DOC_loss_ was lower at colder incubation temperatures (mean *Q*_10_=1.21±0.07), but was not significantly different with respect to the mean Δ^14^C value of DOC_loss_ (paired *t-*test, *P*>0.1; Fig. 4a). Bioenergetic constraints from lower water temperatures therefore did not consistently alter the DOC age or fraction utilized, implying that selective DOC loss continues during colder fall and winter periods, when greater contributions of older DOC could be expected^17^,^18^. Similarly, inorganic nutrient concentration did not significantly influence Δ^14^C values of OC_loss_ (paired *t-*test, *P*>0.1; Fig. 4b), indicating that stoichiometric differences cannot account for the observed downstream trends. Individual water samples displaying differences in the age of DOC utilized at colder temperatures, or under nutrient-replete conditions, all resulted in the loss of an older (^14^C**-**depleted) DOC fraction. This implies that our results represent conservative estimates of permafrost OC loss along the aquatic continuum under our experimental conditions (Fig. 3a,b).

## Discussion

Together, our findings indicate that terrestrial permafrost OC export is already underway and actively contributing to DOC turnover in Arctic fluvial networks. The presence of permafrost OC has previously gone undetected in fluvial networks due to the preferential loss of permafrost OC removing headwater signatures dominated by permafrost inputs^31^, combined with the historical dominance of field sampling on larger main-stem rivers^15^,^32^. In addition, the persistence of modern DOC sourced from vegetation and surface soils causes permafrost OC dynamics to be effectively masked when examining bulk initial OC alone^33^.

The composition of DOC in Arctic stream and river networks is expected to shift under future climate change scenarios^1^,^2^. Permafrost thaw is promoting greater groundwater and sub-surface flow from Arctic and sub-arctic watersheds, likely resulting in the export of DOC characterized by lower-molecular weight compounds and a lower degree of aromaticity^3^,^4^,^17^,^27^,^31^. Across our sites, we observed a linear increase in the proportion of bioavailable carbon (DOC_loss_) with decreasing DOC aromaticity as evidenced by lower SUVA_254_ values^34^,^35^ (*r*^2^=0.50, *P*<0.001; Supplementary Fig. 2), suggesting that increasing future permafrost contributions may promote enhanced microbial metabolism within Arctic fluvial networks. Furthermore, the microbial utilization of older DOC fractions was observed with decreasing OC aromaticity (Fig. 4c) and increasing DOC_loss_ (*r*^2^=0.37, *P*<0.001), suggesting that DOC compositional changes will result in greater ancient carbon turnover. Heterotrophic metabolism and respiration is dependent on low-molecular weight DOC from thawing permafrost soils^24^,^28^. The lower aromatic content and molecular weight of permafrost-derived DOC may efficiently support bacterial production and favour higher carbon-use efficiencies^36^.

The microbial uptake of highly biologically available permafrost carbon can result in the preferential loss of ancient OC in aquatic networks, and the selective export of modern DOC to the ocean. Similar trends in the processing of permafrost-derived DOC among fluvial networks of other major arctic rivers appear likely. All arctic watersheds are warming^6^ and exhibit similar trends and controls upon DOC export^32^. Freshwater discharge is also strengthening from at least the Eurasian arctic watersheds^37^,^38^. Yedoma permafrost in Alaska has also recently been shown to be highly bioavailable in good agreement with studies in Siberia^19^,^24^,^39^,^40^. Additional mechanisms, such as the selective burial of older aged OC, changing microbial community composition or preferential photodegradation of ancient OC may also play an important or complementary role in controlling permafrost turnover in northern high-latitude systems^10^,^12^,^41^. Photodegradation has been shown to influence surface water DOC processing in the Arctic^41^, yet it is currently unclear how sunlight affects the bulk age of DOC. Recognizing that modern bulk DOC signatures in Arctic rivers may disguise a rapidly cycling old C fraction, future efforts to detect the impacts of permafrost thaw should focus on headwater systems where the rapid removal and preferential biological utilization of ancient permafrost-derived DOC appears prevalent. As permafrost thaw accelerates^1^,^6^,^8^, it seems apparent that this will be accompanied by an increase in the amount of bioavailable DOC in aquatic networks, with aged DOC accounting for increased subsidies to aquatic microorganisms ultimately fuelling a positive feedback on climate.

## Methods

### Study area and sample collection

Surface water samples were collected directly from streams and rivers on several expeditions in 2012 and 2013 (Supplementary Table 1). Sampling concentrated during the months of August to October, when surface active layers are deepest and thus maximum export of permafrost-derived OC to inland waters is expected. Additional sampling soon after the spring ice-out period (June) provided insights during a period of minimal surface active layer thaw. We grouped sites by stream width, separating streams (<1–5 m), minor (>5 to ≤200 m) and major tributaries (>200 m), and the Kolyma main stem (Fig. 1). A small proportion of highly turbid (>100 nephelometric turbidity units (NTUs)) upland streams visibly affected by active mechanical soil erosion were examined independently (erosion streams) from unaffected streams (<100 NTU), thus providing more robust source determination^26^ (see the Isotope mixing model section). These streams were always small (<3 m width) and situated within active thermokarst zones with mixed vegetation covered and exposed yedoma banks.

Stream temperature was measured in the field (YSI multi-meter) and sample turbidity immediately upon return to the laboratory (Hach 2100Q). Waters were filtered through pre-combusted glass fibre filters (0.7 μm) on the day of collection using a combusted glass filtration system. After filtration, waters were immediately transferred into pre-combusted amber glass vials (40 ml vials, ∼35 ml water) leaving a headspace. Triplicate aliquots for initial DOC concentration were immediately acidified (trace element grade HCl, ≤pH 2) and stored until analysis. Aliquots for δ^13^C and Δ^14^C were immediately stored frozen (−20 ^°^C) in pre-leached, acid-cleaned HDPE bottles.

### DOC and bioincubations

DOC was calculated as the mean of between three and five injections on a Shimadzu TOC-V where the coefficient of variance across measurements was <2%. Triplicate bioincubation DOC measurements were typically within <5% of each other. The bioavailability of DOC (DOC_loss_) was determined as the difference in triplicate measures of DOC before and after a 28-day laboratory incubation at 20±0.5 ^°^C or 4±1 ^°^C containing the indigenous microbial community from each site. The absorbance of water at 254 nm was measured (Shimadzu UV-1800) and the specific ultraviolet absorbance (SUVA_254_) determined as a proxy for aromaticity^34^. After bioincubation, waters were refiltered as above, and samples collected as before for DOC, δ^13^C and Δ^14^C analyses. Parallel bioavailability incubations conducted with nutrient-amended waters were supplemented with inorganic nitrate and phosphate (KNO_3_/KH_2_PO_4_) according to Redfield ratio, resulting in nutrient-replete incubations in relation to ambient site nutrient concentrations. We used analysis of variance to test between site means and paired *t*-tests to compare parallel incubations results. All tests including Univariate regression and exponential fits were conducted in SPSS21 (IBM).

### Stable and radiocarbon isotope analyses

δ^13^C analyses were conducted at the University of California, Davis Stable Isotope Facility and ^14^C analyses at the Eidgenössiche Technische Hochschule (ETH) Zürich. ^13^C-DOC samples were analysed using an O.I. Analytical Model 1010 TOC analyzer (precision of ±0.2‰) interfaced to a PDZ Europa 20–20 IRMS (Sercon Ltd). ^13^C-DOC measurements were calibrated against the δ^13^C values of KHP and IHSS Suwannee River humic acid in Milli-Q. Waters for ^14^C-DOC analyses were freeze-dried (Christ Alpha 2-4, LSC with a low-carbon vacuum hybrid pump, Vacubrand RC-6; Martin Christ, Labex Instrument AB, Sweden) directly in pre-combusted (850 ^°^C/5 h) quartz tubes. Samples were acidified under HCl vapours to remove carbonates and flame sealed with pre-combusted CuO under vacuum. CO_2_ was cryogenically captured and quantified (∼30 μg carbon) before measurement using a microscale radiocarbon dating system (MICADAS; http://www.ams.ethz.ch/instruments/micadas) and gas-feeding system^42^,^43^. Combusted NIST SRM 4990C oxalic acid was used as a standard for normalization, and blanks were determined using radiocarbon-free CO_2_ both at a concentration of 5% CO_2_ in He. The modern oxalic acid standard was measured to better than 1% relative error and until samples were fully consumed.

Radiocarbon contents are reported as Δ^14^C (‰) and ^14^C age. All radiocarbon values are corrected for procedural blanks with the errors propagated. The isotopic signature (Δ^14^C and δ^13^C) of the utilized DOC fraction (DOC_loss_) was calculated using triplicate measurements of initial DOC (DOC_initial_) and final DOC concentrations (DOC_final_) alongside the associated change in isotopic composition using simple mass balance (equation (1)). Individual errors associated with multiple isotope measurements were propagated to assess error on δ^13^C and Δ^14^C_utilized_.


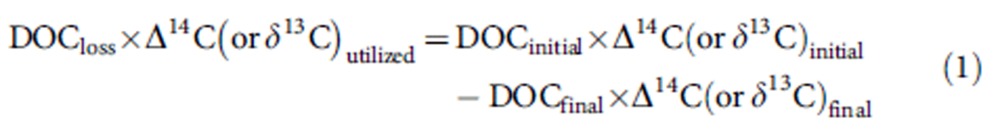


### Isotopic mixing model

Feasible contributions of three end-member sources (permafrost DOC, contemporary DOC and *in situ* autochthonous DOC) to DOC_loss_ at sites were calculated using a dual-carbon isotope mixing model solved using a Monte Carlo simulation approach. Monte Carlo simulations were conducted using the MIXSIAR package^26^ v2.1.2 (https://github.com/brianstock/MixSIAR/releases) within the R programming environment (http://www.R-project.org). MixSIAR is a Bayesian mixing model^44^ that allows the contributions of source end-members to isotope data to be estimated, taking into account the uncertainty in source values^45^ and model error^46^ (residual and process errors). A dual-isotope approach (^14^C/^13^C) was used to increase the accuracy of contribution estimates and to solve for three potential end-member sources.

We defined two separate terrestrial end-members to differentiate between contemporary OC pools (vegetation, surface soils and surface active layer) and permafrost-derived OC that is delivered to freshwaters via ground collapse, or thermokarst processes, bank erosion or deep groundwater flow. An *in situ*-derived DOC source from aquatic primary producers (for example, periphytic algae) was also included. End-member values of each DOC source were determined as outlined below with final mean values and ranges defined as follows: permafrost δ^13^C −26.3±1.3‰, Δ^14^C −940±84‰; contemporary δ^13^C −28.5±2.0‰, Δ^14^C 71.6±170‰; *in situ* autochthonous δ^13^C −19.8±3.0‰, Δ^14^C −78.0±170‰. Error estimates on end-member sources incorporated the entire variance in source values, enabling MixSIAR to fit the most viable end-member source to the model fit^26^. To account for potential fractionation of δ^13^C, the MIXSIAR package was run including discrimination factors set to ±1‰. No fractionation was allowed for Δ^14^C values.

Each experiment was treated as an individual ‘random' event, and site type (yedoma thaw, erosion stream, stream, minor and major tributary and main-stem waters) was included as a ‘random' effect^47^, which improved model fit (determined as a reduction in the Akaike information criterion). Site type (*σ*site=7.1) explained a greater variation in source than individual information alone (*σ*ind=1.8), indicating that the total variation in the source of DOC fuelling microbial demand was driven by location along the fluvial network. Here we present posterior mean contribution estimates (±s.e.m) among sites (Table 1; Supplementary Table 2).

### Isotopic end-member determination

The contemporary δ^13^C (−28.5±2.0‰; *n*=14) and Δ^14^C (71.6±150‰; *n*=21) was derived from literature^18^ and unpublished data (http://arcticgreatrivers.org/data, Vonk *et al.*, unpublished;Supplementary Table 3) collected during the months of May and June from a range of small streams and rivers from the Kolyma River Basin. Data were limited to the early spring period, when water flow is restricted to surface horizons and thus most likely contains a topsoil and vegetation signature. A larger error estimate was applied to the Δ^14^C end-member values to incorporate soil pore water-derived DOC that can be highly ^14^C enriched^25^.

The δ^13^C value of the permafrost end-member (−26.3±1.3‰; *n*=374) is taken from a recent review of Yedoma deposits across Siberia^48^ and was also recently used in a similar dual-carbon isotope model to determine relative contributions of Yedoma to the East Siberian Arctic Shelf^49^. The value closely aligns with other δ^13^C DOC values (−25.7±0.3‰; *n*=7) measured in Yedoma thaw waters^19^ and soils (−25.1±0.3‰; *n*=2) from the Kolyma River Basin (>2 m)^50^. The Δ^14^C value for permafrost OC was taken from a synthesis of Yedoma data as detailed in Vonk *et al.* (2012; Supplementary Table S4 and references herein)^49^. This value (−940±84‰; *n*=300) closely aligns with Yedoma permafrost thaw water DOC measured in the Kolyma River Basin^19^,^33^ (−944±23‰; *n*=6). By using a maximum estimate for the old carbon permafrost end-member, we ensure conservative estimates of old C supply to stream and river waters.

The δ^13^C value of aquatic primary producers (for example, periphytic algae) is highly variable and difficult to assess, with literature values varying from −40 to −10‰^51^. δ^13^C end-member values were estimated using dissolved inorganic carbon measurements from the Kolyma River (Raymond *et al.*, unpublished) (−7.5 to −7.2‰) and Lena River (−4.0 to −3.5‰), and periphytic OC δ^13^C (−23.0 to −16.6‰) calculated using an established relationship between δ^13^C of DIC and of epilithic algae from across 70 different streams and rivers spanning arctic and temperate environments^52^. This linear relationship was derived from direct measurements of herbivore δ^13^C and DIC δ^13^C values^52^ and demonstrated that a consistent fractionation (for example, 19 or 20‰ assumed fixed photosynthetic fractionation) did not accurately constrain δ^13^C of algae from DIC measurements in lotic systems. This resulted in a relative fractionation for this study ranging from 13.1 to 15.5‰. Our calculated δ^13^C for algae closely align with values measured in a single plankton bloom on the Pantileikha River within the Kolyma Basin of −23.3‰ (ref. 53) and of the end-members calculated for marine OC in Vonk *et al.*^49^ of −24‰. Both values lie within our end-member estimates once the model discrimination factor of up to 1‰ has been applied for δ^13^C fractionation. Phytoplankton should reflect the current Δ^14^C of DIC in waters. Δ^14^C of DIC from the Kolyma River ranged from −77.0 to −79.0‰ (Raymond *et al.*, unpublished), and was therefore adopted as the mean end-member value in the mixing model.

## Additional information

**How to cite this article:** Mann, P. J. *et al.* Utilization of ancient permafrost carbon in headwaters of Arctic fluvial networks. *Nat. Commun.* 6:7856 doi: 10.1038/ncomms8856 (2015).

## Supplementary Material

Supplementary Information Supplementary Figures 1-2, Supplementary Tables 1-3 and Supplementary References.

## Figures and Tables

**Table 1:** DOC and isotope characteristics throughout the fluvial network.

	***n***	**Initial DOC (μM)**	**SUVA**_**254**_ **(l mgC**^**−1**^ **m**^**−1**^**)**	**DOC**_**loss**_ **(**%**)**	**Init. Δ**^**14**^**C (‰)**	**OC**_**loss**_ **Δ**^**14**^**C (‰)**	**Mean OC**_**loss**_ **age (**^**14**^**C years)**	**Permafrost** **(**%**)**	**Contemporary** **(**%**)**	***In situ*** **(**%**)**
Yedoma thaw	5	10,939±1,278	1.63±0.19	47.2±7.6	−884±41	−873±48	16,576	97.2±0.8	1.7±0.5	1.1±0.5
Erosion streams	3	2,503±518	3.25±0.44	17.5±4.9	−214±145	−405±185	4,171	42.8±20.7	56.4±20.7	0.8±0.4
Streams	25	1,691±87.6	3.44±0.11	13.3±2.0	51±6	−21±50	170	13.0±4.0	85.9±4.0	1.1±0.2
Minor tributaries	7	766±110	3.56±0.19	21.0±5.5	25±13	26±88	Modern	5.7±3.5	93.7±3.6	0.6±0.2
Major tributaries	8	511±68	4.03±0.08	12.2±1.8	40±3	68±32	Modern	0.6±0.1	73.1±8.4	26.3±9.9
Main stem	6	425±46	3.67±0.10	14.6±1.8	22±9	74±42	Modern	0.7±0.1	88.6±2.8	10.7±4.4

DOC, dissolved organic carbon; OC, organic carbon.

Mean initial DOC concentration, specific ultraviolet absorbance at 254 nm (SUVA_254_), percent DOC loss over 28 days (DOC_loss_), calculated Δ^14^C value and mean radiocarbon age of DOC_loss_ (using equation (1) in Methods). The sample size at each site type is provided (*n*) and individual data are detailed in Supplementary Table 2. Mean percentage (±s.e.m.) contribution of permafrost, contemporary and *in situ*-derived DOC to DOC_loss_ determined using the dual-carbon isotope mixing model.
