# Supplementary material for: Utilization of ancient permafrost carbon in headwaters of Arctic fluvial networks
Source: Nat Commun. 2015 Jul 24;6:7856. doi: 10.1038/ncomms8856 (PMC4525200; doi:10.1038/ncomms8856)
Supplement: Supplementary Information — Supplementary Figures 1-2, Supplementary Tables 1-3 and Supplementary References. [file ncomms8856-s1.pdf]

Supplementary Figures

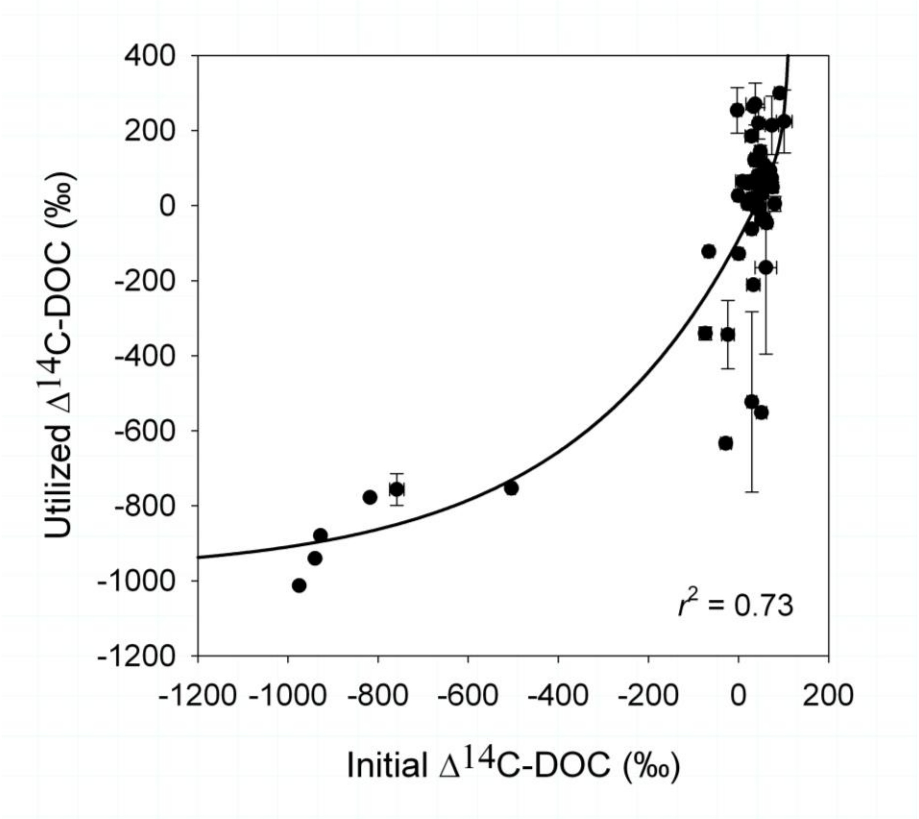

Supplementary Figure 1.

Relationship between $\Delta^{14}C$ values of initial and utilized DOC across incubation waters. Error bars represent the propagated SEM error for $\Delta^{14}C$ estimates. The non-linear nature of the relationship suggests that small increases in the initial age of DOC result in relatively large increases in the age of the utilized DOC pool. Bold line represents an exponential growth fit (y0+a*exp(b*x)+c*exp(d*x)) (r^2 = 0.73; p <0.0001; n =51).

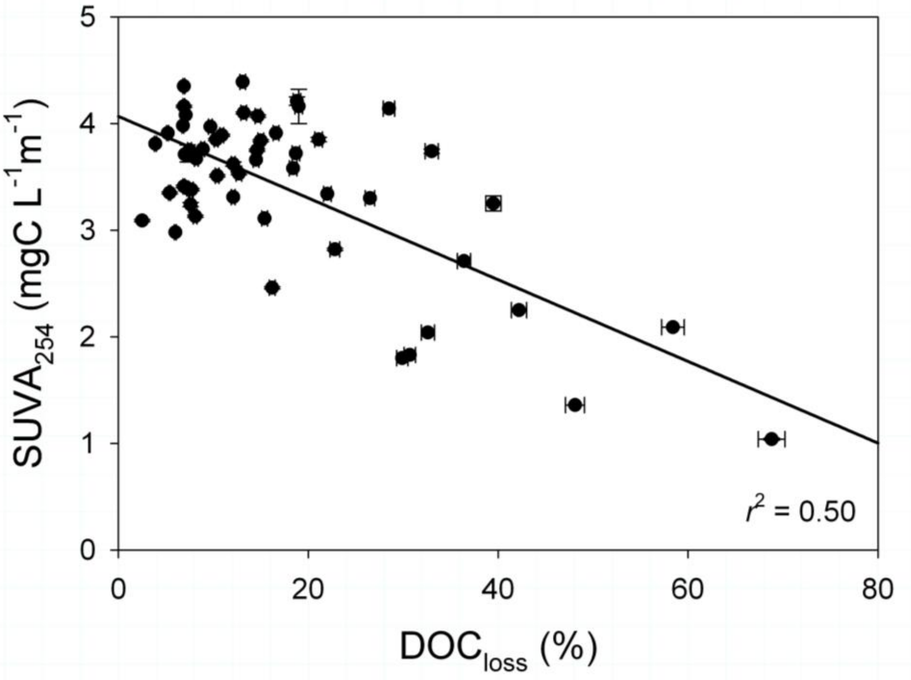

Supplementary Figure 2.

The relationship between DOC_{loss} and initial $SUVA_{254}$ values across incubation waters. $SUVA_{254}$ provides a unique proxy of DOC aromaticity[1,2]. As DOC aromaticity decreased in freshwaters, greater proportions of the DOC were utilized under identical bioincubation conditions. Bold line represents a linear regression fit ($r^2 = 0.50$, $P < 0.001$, $n = 53$)

Supplementary Tables

Supplementary Table 1

Latitude and longitude in decimal degrees of each sampling site with type.

Type	Latitude dd.dd	Longitude dd.dd
Main-stem	68.7211	158.6802
Major trib.	68.6887	158.5985
Stream	68.6291	159.1793
Erosion Streams	68.6298	159.1330
Yedoma thaw	68.6305	159.1501
Stream	68.7432	161.4130
Minor trib.	68.6767	161.4204
Stream	68.7022	161.5197
Stream	68.7104	161.5102
Stream	68.7244	161.4736
Stream	68.6700	161.7831
Major trib.	68.4591	160.7828
Major trib.	68.4519	160.8121
Minor trib.	69.4916	161.8413
Minor trib.	68.9265	161.6571
Stream	69.4654	161.7636
Stream	69.4406	161.7066
Minor trib.	68.7074	161.5091
Stream	68.7592	161.4476
Main-stem	68.6285	161.2131

.

Supplementary Table 2.

Individual sampling dates and type with associated DOC characteristics

Measured DOC concentration, DOC loss over 28 days (20°C; DOC_{loss}), initial $\delta^{13}C$, $\Delta^{14}C$ and utilized $\delta^{13}C$, $\Delta^{14}C$ values calculated using equation 1 (see methods). Percent contribution of permafrost OC (Perm.), contemporary OC (Cont.) and *In situ*–derived OC (In-situ) to DOC_{loss} are estimated using a dual-carbon-isotope mixing model. Specific UV absorbance (SUVA) at 254 nm as a proxy for aromaticity[1,2]. Temperature (Temp.) and turbidity (Turb., in nephelometric turbidity units (ntu)). Tubidity over maximum (max) > 1000 NTU. n/a represents parameter not measured. Samples in bold were not included in mixing model analyses due to missing values.

Sampling Date	Type	DOC	DOC_{loss}	Initial (‰)		Utilized (‰)		% cont. to DOC_{loss}			$SUVA_{254}$	Temp.	Turb.
		µM	%	$\Delta^{14}C$	$\delta^{13}C$	$\Delta^{14}C$	$\delta^{13}C$	Perm.	Cont.	In-situ	mgC $L^{-1}m^{-1}$	°C	ntu
26/08/12	Main-stem	241	21.1	-0.8	-26.1	26.6	-25.5	0.8	89.8	9.3	3.9	11.3	21
26/08/12	L. Tributary	335	6.8	29.7	-26.4	-62.6	-23.9	1.2	65.7	33.1	4.0	9.0	8
26/08/12	Stream	1063	7.7	33.2	-28.5	263.8	-26.3	2.3	97.2	0.5	3.4	7.7	6
26/08/12	Erosion Stream	3527	16.2	-503.8	-27.1	-753.3	-25.4	81.3	17.9	0.8	2.5	3.5	354
27/08/12	Yedoma thaw	15793	48.1	-817.9	-25.9	-777.8	-25.7	95.6	2.7	1.6	1.4	1.3	max.
27/08/12	Stream	1607	2.5	57.8	-28.2	106.8	-23.3	3.5	94	2.4	3.1	4.3	13
28/08/12	S. Tributary	591	10.4	-2.6	-28.3	253.8	-28.1	1.1	98.4	0.4	3.5	10.3	6
28/08/12	Stream	2157	6.0	36.4	-28.2	120.0	-23.4	3.3	94.3	2.4	3.0	4.5	2
28/08/12	Stream	1480	8.1	69.6	-28.4	94.1	-26.4	4.1	95.1	0.8	3.1	5.2	9
28/08/12	Stream	2559	12.1	61.1	-28.6	61.7	-28.5	4.3	95.2	0.4	3.3	5.5	6
31/08/12	Stream	1506	13.2	0.3	-28.3	-128.4	-27.5	12.4	86.8	0.8	4.1	n/a.	13
31/08/12	L. Tributary	756	10.9	43.4	-28.7	80.4	-24.4	0.6	77.1	22.3	3.9	7.6	10
31/08/12	L. Tributary	554	14.6	36.1	-28.3	122.2	-27.4	0.5	90.8	8.7	3.8	8.0	6
31/08/12	Mainstem	468	12.7	21.0	-27.8	58.8	-22.9	0.7	79.2	20.1	3.5	9.8	22
03/09/12	S. Tributary	676	12.1	44.4	-28.5	219.2	-27.9	1.3	98.3	0.4	3.6	5.7	7
03/09/12	S. Tributary	893	8.9	48.1	-28.4	45.1	-27.6	2.4	97	0.6	3.8	9.0	7
03/09/12	Stream	1360	18.7	31.1	-29.1	12.0	-28.7	5.1	94.4	0.5	3.7	5.3	5

Sampling Date	Type	DOC µM	DOC_{loss} %	Initial (‰) $\Delta^{14}C$	Initial (‰) $\delta^{13}C$	Utilized (‰) $\Delta^{14}C$	Utilized (‰) $\delta^{13}C$	% cont. to DOC_{loss} Perm.	% cont. to DOC_{loss} Cont.	% cont. to DOC_{loss} In-situ	$SUVA_{254}$ mgC $L^{-1}m^{-1}$	Temp. ^oC	Turb. ntu
03/09/12	Stream	1769	28.5	50.4	-28.8	29.4	-28.8	4.7	94.9	0.5	4.1	6.3	2
10/09/12	Stream	2620	32.6	20.5	-28.1	4.5	-27.8	5.7	93.5	0.7	2.0	1.3	3
10/09/12	S. Tributary	854	36.4	28.3	-28.3	19.0	-28.3	2.6	96.7	0.7	2.7	6.9	3
10/09/12	Stream	1649	42.2	73.5	-28.4	73.4	-28.0	4.1	95.4	0.5	2.2	1.3	5
12/09/12	Stream	1778	18.4	58.9	-28.8	71.0	-28.1	4.2	95.4	0.5	3.6	1.1	2
14/09/12	Main-stem	362	7.6	8.8	-28.0	64.3	-23.2	0.7	81.1	18.3	3.2	6.5	14
14/09/12	Erosion Stream	2123	26.5	-65.9	-29.1	-122.3	-28.7	10.4	89	0.6	3.3	1.5	162
14/09/12	Yedoma thaw	8391	30.7	-758.6	-26.0	-756.6	-25.1	95.0	2.9	2.1	1.8	1.3	max.
15/09/12	Stream	2461	22.8	62.6	-28.4	-46.3	-28.0	7.3	92.1	0.6	2.8	0.3	10
26/06/13	L. Tributary	218	19.0	45.1	-27.2	125.4	-23.9	0.5	75.7	23.8	4.2	11.6	2
**26/06/13**	**L. Tributary**	**552**	**13.1**	**-18.3**	**-28.5**	**n/a.**	**-27.8**	**n/a.**	**n/a.**	**n/a.**	**4.4**	**n/a.**	33
26/06/13	L. Tributary	368	18.8	28.9	-28.2	184.6	-25.6	0.4	87.1	12.4	4.2	13.4	30
26/06/13	Yedoma thaw	9579	68.8	-974.9	-26.2	-1013.1	-26.1	99.0	0.6	0.4	1.0	n/a.	max.
28/06/13	Stream	2094	22.0	39.8	-28.4	-7.7	-28.3	6.0	93.5	0.5	3.3	6.4	2
30/06/13	S. Tributary	362	39.5	-23.5	-27.9	-343.9	-27.8	23.2	75.9	0.9	3.2	11.3	6
30/06/13	S. Tributary	1295	33.0	57.8	-28.4	-34.8	-28.1	3.6	95.8	0.6	3.7	14.9	3
**30/06/13**	**Stream**	**1331**	**15.4**	**n/a.**	**-28.2**	**n/a.**	**-26.6**	**n/a.**	**n/a.**	**n/a.**	**3.1**	**n/a.**	**n/a.**
01/09/13	Yedoma thaw	10866	58.4	-940.0	-26.0	-940.2	-25.4	98.6	0.9	0.6	2.1	n/a.	max.
05/09/13	Stream	1865	7.0	80.1	-28.0	4.5	-22.3	5.1	88.4	6.4	3.7	1.4	5
05/09/13	Stream	1363	3.9	74.3	-28.8	213.7	-28.3	2.5	97.1	0.4	3.8	2.9	1
**05/09/13**	**S. Tributary**	**694**	**6.9**	**37.1**	**-27.6**	**n/a.**	**n/a.**	**n/a.**	**n/a.**	**n/a.**	**4.4**	**n/a.**	45
05/09/13	Stream	2164	7.1	29.6	-28.8	-523.0	-27.9	56.3	43.1	0.6	4.1	2.9	1
09/09/13	L. Tributary	567	7.5	48.4	-27.6	31.5	-19.2	0.5	26.6	72.8	3.7	7.1	15
09/09/13	L. Tributary	741	6.9	47.2	-27.7	-4.4	-27.3	0.8	88.4	10.8	4.2	7.0	8
09/09/13	Stream	1029	8.2	48.2	-28.2	144.6	-27.4	3.3	96.3	0.5	3.7	5.3	6
09/09/13	Erosion Stream	1858	9.7	-73.8	-28.3	-340.6	-26.7	36.7	62.4	0.9	4.0	2.8	199

Sampling Date	Type	DOC µM	DOC_{loss} %	Initial (‰) $\Delta^{14}C$	Initial (‰) $\delta^{13}C$	Utilized (‰) $\Delta^{14}C$	Utilized (‰) $\delta^{13}C$	% cont. to DOC_{loss} Perm.	% cont. to DOC_{loss} Cont.	% cont. to DOC_{loss} In-situ	$SUVA_{254}$ mgC $L^{-1}m^{-1}$	Temp. ^{o}C	Turb. ntu
09/09/13	Main-stem	450	16.6	37.0	-27.8	270.6	-25.2	0.3	94	5.7	3.9	7.3	5.6
09/09/13	Yedoma thaw	10066	29.9	-927.9	-26.0	-879.4	-25.5	97.9	1.2	0.9	1.8	0.8	max.
10/09/13	Stream	1409	5.2	60.7	-28.2	-164.9	-25.3	17.0	81.1	1.8	3.9	3.0	1
10/09/13	Stream	1664	6.9	101.5	-28.0	224.1	-26.8	2.5	97	0.5	3.4	1.6	2
12/09/13	Main-stem	572	14.5	51.3	-27.5	-37.7	-27.2	1.2	92.9	5.9	3.7	5.7	6
12/09/13	Stream	1510	10.3	91.7	-28.2	299.5	-23.1	1.9	97	1.1	3.8	3.6	1
12/09/13	Stream	1674	5.4	51.0	-28.1	-551.8	-26.6	60.9	38.3	0.8	3.3	1.6	1
19/09/13	Main-stem	455	15.0	31.7	-27.7	65.5	-27.3	0.7	94.4	4.9	3.8	5.6	4.5
19/09/13	Stream	1345	14.7	-28.1	-28.1	-633.6	-26.7	69.3	30	0.7	4.1	3.4	2
19/09/13	Stream	1627	7.3	75.3	-28.2	49.3	-25.3	4.9	94	1.1	3.4	2.9	3
05/10/13	Stream	1193	7.3	33.0	-28.3	-211.1	-25.5	21.3	77.1	1.6	3.7	0.2	1

Supplementary Table 3.

Literature and unpublished $\delta^{13}C$ and $\Delta^{14}C$ values for contemporary DOC end-member.

PARTNERS data are available at http://arcticgreatrivers.org/data. Unpublished data from Vonk *et al.* were collected as part of the POLARIS project http://www.thepolarisproject.org. Table contains data from May and June only when water flow is restricted to surface horizons and thus most likely contains a topsoil and vegetation signature.

Site Type	$\delta^{13}C$ -DOC (‰)	$\Delta^{14}C$-DOC (‰)	Reference
Stream	-28.4	149.8	[3]
Stream	-30.2	81.5	[3]
Main-stem	-30	116.1	[3]
Stream	-28.9	51.7	[3]
Main-stem	-32.2	52.6	[3]
Stream	-27.98	108.4	[3]
Stream	-27.5	107.4	[3]
Stream	-28.3	69.6	[3]
Major Tributary	-	67.8	[3]
Major Tributary	-27.2	56.2	[3]
Mainstem	-27.9	52.7	[3]
Mainstem	-27.3	86.8	PARTNERS
Mainstem	-30.0	-3.9	PARTNERS
Main-stem	-27.4	44.8	PARTNERS
Stream	-	119.5	Vonk et al, unpublished
Stream	-	118.3	Vonk et al, unpublished
Main-stem	-	77.0	Vonk et al, unpublished
Main-stem	-26.1	-12.7	PARTNERS
Main-stem	-	40.4	Vonk et al, unpublished
Stream	-	61.6	Vonk et al, unpublished
Stream	-	58.6	Vonk et al, unpublished
Mean	-28.5	71.6	

Supplementary References

1. Spencer, R. G. M., Aiken, G. R., Wickland, K. P., Striegl, R. G. & Hernes, P. J. Seasonal and spatial variability in dissolved organic matter quantity and composition from the Yukon River basin, Alaska. *Global Biogeochem. Cycles* **22,** GB4002 (2008).
2. Weishaar, J. L. *et al.* Evaluation of Specific Ultraviolet Absorbance as an Indicator of the Chemical Composition and Reactivity of Dissolved Organic Carbon. *Environ. Sci. Technol.* **37,** 4702–4708 (2003).
3. Neff, J. C. *et al.* Seasonal changes in the age and structure of dissolved organic carbon in Siberian rivers and streams. *Geophysical Research Letters* **33,** L23401 (2006).
